# Multi-scale interplays of biotic and abiotic drivers shape mammalian sub-continental diversity over millions of years

**DOI:** 10.1038/s41598-018-31699-6

**Published:** 2018-09-07

**Authors:** Juan L. Cantalapiedra, M. Soledad Domingo, Laura Domingo

**Affiliations:** 1 Museum für Naturkunde, Leibniz-Institut für Evolutions und Biodiversitätsforschung, Invalidenstraße 43, 10115 Berlin, Germany; 2 Dpto Ciencias de la Vida, Universidad de Alcalá, 28805 Alcalá de Henares, Madrid Spain; 3 Dpto Ecología Evolutiva, Estación Biológica de Doñana (CSIC), Américo Vespucio 26, 41092 Sevilla, Spain; 4 Dpto Geodinámica, Estratigrafía y Paleontología, Facultad de Ciencias Geológicas, Universidad Complutense de Madrid, José Antonio Nováis 12, 28040 Madrid, Spain; 5 Dpto Geología Sedimentaria y Cambio Medioambiental, Instituto de Geociencias (CSIC, UCM), Severo Ochoa 7, 28040 Madrid, Spain; 6 Earth and Planetary Sciences Department, University of California Santa Cruz, 1156 High Street, CA 95064 Santa Cruz, USA

## Abstract

The reconstruction of deep-time diversity trends is key to understanding current and future species richness. Studies that statistically evaluate potential factors affecting paleodiversity have focused on continental and global, clade-wide datasets, and thus we ignore how community species richness build-up to generate large-scale patterns over geological timescales. If community diversity is shaped by biotic interactions and continental and global diversities are governed by abiotic events, which are the modulators of diversity in subcontinental regions? To address this question, we model Iberian mammalian species richness over 13 million years (15 to 2 Ma) using exhaustive fossil evidence for subcontinental species’ ecomorphology, environmental context, and biogeographic affinities, and quantitatively evaluate their impact on species richness. We find that the diversity of large Iberian mammals has been limited over time, with species richness showing marked fluctuations, undergoing substantial depletions as diversity surpasses a critical limit where a significant part of the niches is unviable. The strength of such diversity-dependence has also shifted. Large faunal dispersals and environmental heterogeneity increased the system’s critical diversity limit. Diversity growth rate (net migration and diversification) also oscillated, mainly modulated by functional saturation, patchiness of canopy cover, and local temperature and aridity. Our study provides quantitative support for subcontinental species pools being complex and dynamic systems where diversity is perpetually imbalanced over geological timescales. Subcontinental diversity-dependence dynamics are mainly modulated by a multi-scale interplay of biotic and abiotic factors, with abiotic factors playing a more relevant role.

## Introduction

Because unveiling past diversification processes is fundamental to understanding current and future diversity patterns^1–3^, the study of deep-time diversity trends —and its drivers and limiters— is a major task in paleobiology and macroecology. Factors shaping diversity through time probably vary with geographic and temporal scales^4–7^. For example, global clade growth dynamics at the large spatial and temporal scales show patterns compatible with a process where the strength of diversity-dependence tracks global climate^8^. At the continental scale, paleontological evidence suggests that diversity might be constrained^9–12^, with stasis resulting from a sequential replacement of species and clades^7,11^ where competition may play an important part^13^. Most of studies tackling these issues have been conducted at such large scales —i.e. global, clade-wise or continental^6,11,14–16^—, and, despite several studies focusing on diversity dynamics within smaller regions —i.e. subcontinental regional pools^17–19^—, these do not statistically test for diversity limits and do not address the role of modulating factors (e.g. climate, competition, etc.) jointly with diversity models in a quantitative framework. Thus, it is still unclear whether diversity is bounded in such smaller regions over evolutionary time, and, if so, what are its limiters. If diversity at the smallest temporal and spatial scale —i.e. local community— is mainly ruled by biotic interactions^4,20^, how do these processes build-up to render the patterns observed at the largest spatial scales?

Addressing this question with paleontological data is challenging, because the quantity of such information decreases with spatial scale^7,21^. Furthermore, if we are to evaluate and model the role of factors potentially impacting sub-continental diversity (e.g. niche saturation, ecological function, environmental shifts, or biogeography), an ideal paleontological record should provide proxies for such factors at the sub-continental scale. Finally, to appropriately capture potential competition and niche saturation dynamics, such a regional fossil dataset should contain information at the species level^6,22^, which demands a well-resolved taxonomy.

We here focus on diversity trends of subcontinental species pools —with emphasis in carrying capacities (*K*), as well as diversification and migration rates (*r*)— drawing information from the exceptional fossil record of the Iberian Neogene, between 15 and 2 Ma. Our investigation draws from exhaustive occurrence and ecomorphological information for over 200 species, and their environmental context (based on over 1100 stacked δ^13^C and δ^18^O records from fossil tooth enamel). Importantly, our approach statistically evaluates the incidence of diversity-dependent processes and the modulating role of seven different factors, including biotic (i.e. ecological disparity and niche saturation) and abiotic (i.e. local and global environmental shifts, biogeographic events) drivers that operate at different geographic scales.

## Results and Discussion

**Figure 1:**
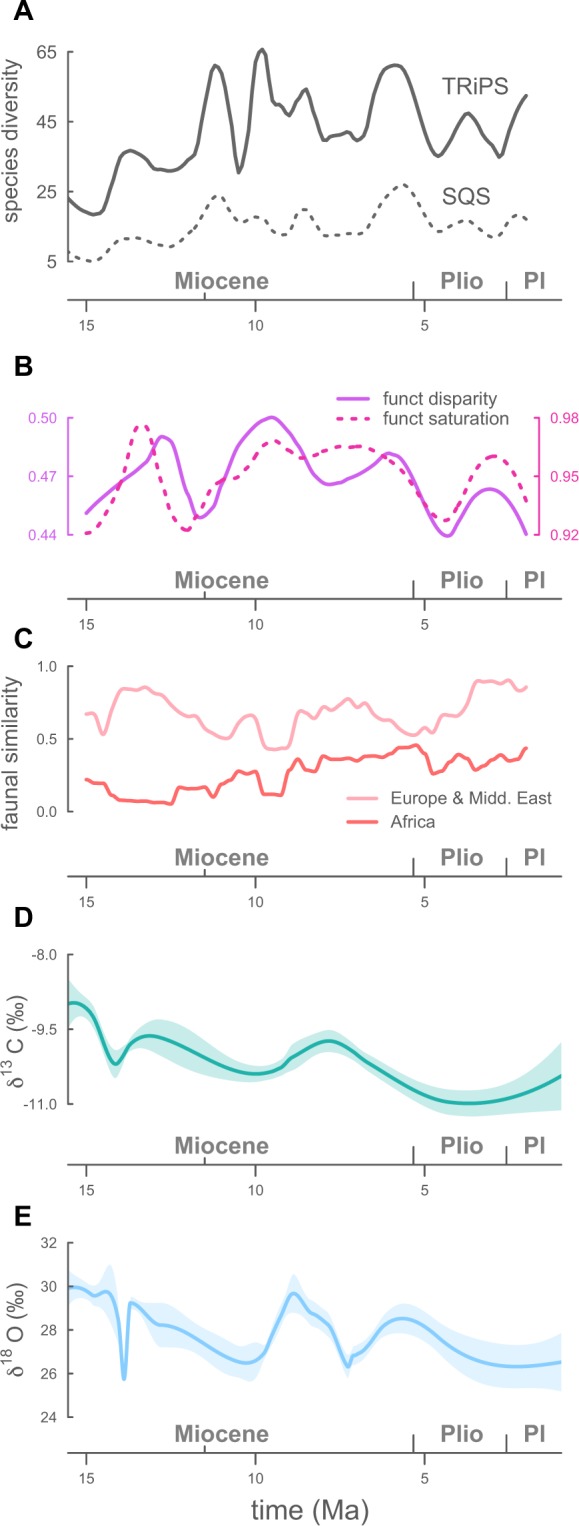
Iberian mammals diversity and analysed factors. (**A**) Iberian mammalian diversity through time estimated using a maximum likelihood method (TRiPS^23^) and a subsampling method (SQS^24^) equal-coverage subsampling (share-holder-quorum)^24^, based on 100 datasets with resampled ages (see Fig. S1). (**B**) Functional disparity and functional saturation (FD and FS) through time. (**C**) Biogeographic affinities of the Iberian Peninsula. (**D**,**E**) Stacked δ^13^C and δ^18^O records from Iberian herbivore fossil tooth enamel. Higher δ^13^C values reflect more open habitats. Higher δ^18^O values reflect warmer environments with more intense evaporation of water bodies. General trends based on local regression fitting (LOESS) and their 95% prediction band is shown. LOESS capture the general trend, reduce the influence of extreme points and the noise caused by the temporal uncertainty in our data. For visual clarity, LOESS curves and point clouds are shown in supplementary figures. See Methods for more details. Plio, Pliocene. Pl, Pleistocene.

We use two different methods for estimating biodiversity through time while controlling for sampling: a maximum likelihood method (True Richness estimated using a Poisson Sampling, or TRiPS^23^), and a subsampling method (the Shareholder Quorum Subsampling method, or SQS^24^) (see supplementary methods and Fig. S1). Both approaches produce very similar diversity trajectories through time (Fig. 1) and congruent results in our modelling approach. Models derived from the TRiPS diversity curve are more clearly discriminated according to their fit (the two best TRiPS-based models aggregate 70% of the support; only 30% in SQS-derived models; see Dataset S1). Thus, unless stated, we mainly discuss the results of our models based on the TRiPS diversity, and highlight incongruences with SQS-based results when necessary.

**Figure 2:**
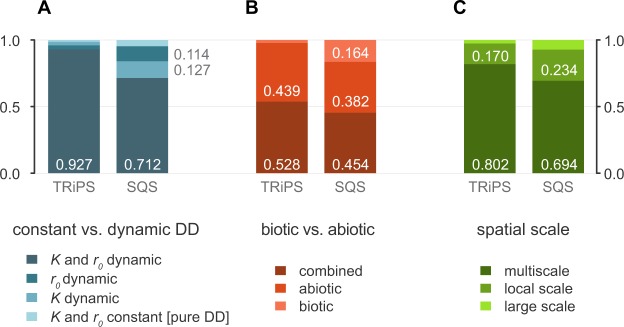
Aggregated AICc-based support for diversity models run on diversity trajectories estimated using TRiPS and SQS. Total support according to temporal changes in the strength of diversity dependence (**A**), the relevance of biotic and abiotic factors (**B**), and the spatial scale of the factors analyzed (**C**). Only values larger than 0.1 are depicted.

After removing sampling effects, the diversity of Iberian large mammals shows marked fluctuations over the last 15 myr (Fig. 1A). Saw-like diversity profiles like these are common in paleontological literature and have been used to argue in favor of unbounded diversity dynamics^22^. Nevertheless, and independently of the method used to reconstruct species richness, the probability of the observed diversity trend being shaped by unbounded competition dynamics is very low: the total support for the ‘damped increase’ model and models without competition is 5% for the TRiPS diversity and 15% for SQS’s (Fig. 2A). Interestingly, we found little support for scenarios where diversity simply tracks biotic and abiotic factors (aggregated support 0.5%), revealing that these alone should not be used as diversity proxies. Rather, we recover a strong signal of saturation dynamics in sub-continental regions over evolutionary timescales. The overall mean support for a finite capping species richness regulated by diversity-dependence, including scenarios where diversity expands and collapses (the ‘expansion and collapse’ model), and where it plateaus around a carrying capacity (the ‘contest’ model; see methods and Dataset S1), was ~95%. In particular, our modelling approach suggests that the diversity dynamics of Iberian mammalian faunas better fit an scenario of ‘expansion and collapse’ (mean support of 81%, 5 times as probable as the ‘contest’ model; Dataset S1) whereby biomass is progressively partitioned in the system as more species are added^25^, eventually surpassing a critical diversity ceiling (*K*) at which point a significant proportion of niches become unviable and the regional species pool undergoes substantial depletion. Thus, over long timescales, regional species pools may mimic the expansion and crunch model predicted and reported for clade-scale dynamics^8,25,26^.

As expected^6^, diversity-dependence (DD) models where *K* and the intrinsic diversification rate (*r*_0_) are fixed (which we term ‘pure DD models’) fail to explain diversity dynamics over long timescales. Rather, we found evidence that the strength of the diversity-dependence at sub-continental scales is constantly reshaped by local environmental changes, large-scale biogeographic events and niche space dynamics. The support for models incorporating substantial shifts in the system’s carrying capacity (*K*) or/and diversification (*r*_0_) is 99% (Fig. 2A).

**Figure 3:**
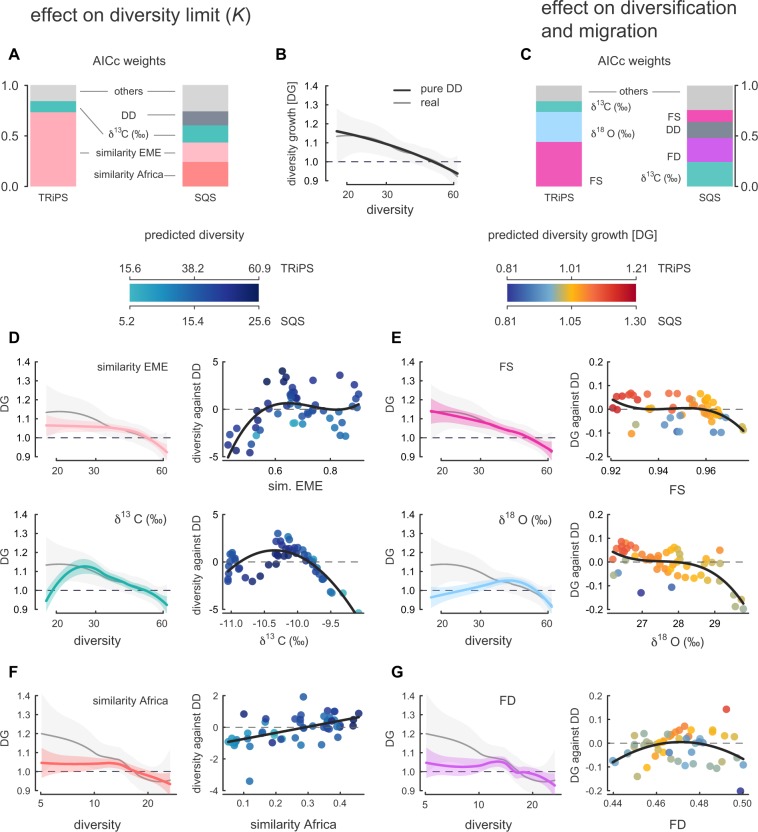
Effect of the most relevant modulating factors on the strength of diversity dependence. Bar-plots (**A**,**C**) show AICc weights aggregated by factors regulating carrying capacity and diversification + migration, respectively, for analyses run on both TRiPS and SQS diversities. Diversity growth (DG; diversity in one bin divided by the diversity in the previous bin) plotted against observed diversity, based on AICc-weight-averaged predicted values from pure DD models (**B**). Light grey represents the real trend. General trends based on local regression fitting (LOESS) and their 95% prediction band is shown. (**D**,**E**) show results from TRiPS diversity. (**F**,**G**) show results only relevant in SQS-based models but also discussed in the main text. (**D**,**F**) Predicted DG averaged from models where *K* is substantially influenced by a factor. The second column in D shows the differences between model averaged predicted diversity from pure DD models and models where each factor regulates *K*, plotted against each factor. Points are colored according to the predicted diversity under each factor (different scale for TRiPS and SQS diversity). Dark lines represent linear or polynomial regressions that are significant (*P* < 0.05). (**E**,**G**) Same as D, but here we average model predictions based on the influence of each factor on *r*_***0***_. The second column shows the differences between model averaged predicted diversity growth from pure DD models and models where each factor regulates *r*_***0***_, plotted against each factor. Results for all the factors are included in Figs S8 and S9. DD, diversity dependence. DG, diversity growth. EME, Europe and the Middle East. FD, functional disparity. FS, functional saturation.

The number of species that the system is able to sustain (carrying capacity, *K*) has substantially changed over evolutionary time (support of 95%; Fig. 2A) and is mainly controlled by continental-scale biogeographic events and shifts in vegetation. In TRiPS-derived models the similarity of Iberian faunas with those from Europe and the Middle East (EME) appears as the main factor controlling *K* (73% of the support) followed by changes in the canopy cover, based on δ^13^C isotopes (11%; Fig. 3A,D). In SQS-derived models, both the similarity with Africa and Europe-Middle East faunas contribute to modulate *K*, together aggregating 43% of the support, and the changes in canopy cover (δ^13^C) contribute with a 17% of the total fit (Dataset S1).

According to the TRiPS-derived models, the isolation of Iberian faunas from the EME region translates into a smaller carrying capacity and less precited diversity than expected under the pure DD scenarios where *K* is constant (Fig. 3). As the EME influence increases, so does *K*, with the richest time windows showing substantially more species than in pure DD scenarios (Fig. 3D). Also, the rate of species accumulation (diversity growth, DG) in EME-modulated *K* models is slower than expected when diversity is low (Fig. 3D). These findings indicate that during geographic isolation and higher endemism, the Iberian mammalian faunas were not as rich as the system would have allowed, with local speciation never contributing to the species packing as dispersals from the main continent did.

When diversity is estimated with SQS, both EME and, especially, African affinities contribute similarly to the diversity cap (Fig. S9). However, the effect of the resemblance with EME is not as evident as in TRiPS-derived models (Fig. S8), and its effect is less significant (Dataset S1). Nevertheless, the influence of Africa on Iberia’s large mammal SQS diversity is clear (Fig. 3A,F). We found a significant correlation between the resemblance with African faunas and *K* (Fig. 3F). Not only did African connections increase Iberian diversity limit beyond the prediction from pure DD models; in moments when Iberian faunas show less African influence, the biogeographic model predicts an smaller *K* than pure biotic models when SQS is used to infer past species richness (Fig. 3F), suggesting a major role of African faunas in Neogene Iberian mammalian diversity^27^. Our metric of biogeographic affinities should reflect certain environmental conditions^18,28^ that likely are not entirely captured by our isotopic data. In fact, the similarity with African faunas is significantly correlated with a denser canopy cover across the analysis interval (correlation with δ^13^C: *P* < 0.001; Kendall’s τ = −0.318). Furthermore, the African immigrants may had contributed to the system with ecomorphological traits beyond those included in our dataset. A combination of these factors could have facilitated the packing of species in the system beyond expectations from pure DD models. In TRiPS-derived models, the similarity with African faunas is also associated with higher diversity than in pure DD models (Fig. S8), but the contribution of the model to the total fit is below 2% (Dataset S1). One potential explanation for this contrasting result is the different diversity trends yielded by TRiPS and SQS for the latest Miocene. SQS identifies a maximum richness around 6 Ma, coincident with the maximum similarity with African faunas, whereas for TRiPS this diversity peak is not the maximum of the analyses interval (Figs 1A and S1). Also, it could be possible that, given the correlation between the African influence and the canopy cover, part of the African signal in the TRiPS-based analyses is recovered by models incorporating δ^13^C-modulated *K* (see below). Although the influence of Africa in Iberian faunas has been previously reported^27^, our understanding of its impact in mammalian taxonomic and ecological diversity in the Iberian Peninsula will benefit from novel quantitative approaches and further investigation.

Shifts in vegetation type and canopy cover (tracked through tooth enamel δ^13^C values) also impacted the number of species that the system was able to sustain (11% and 17% of total support in TRiPS- and SQS-based models, respectively). At the extremes of the δ^13^C values, when habitats are dominated either by grassland or denser woodland^29^, predicted diversity tends to decrease in comparison with fixed-*K* models (Fig. 3D). Intermediate δ^13^C values, reflecting open woodlands or wooded grasslands^29^, slightly increase the predicted diversity over estimates from fixed-*K* models (Fig. 3D). More heterogeneous landscapes are expected to weaken crowding effects, both by increasing speciation rates (see below), and by promoting regional coexistence^22,30,31^. Taken together, these patterns reveal a system where local environmental settings were a secondary modulator of carrying capacity, accommodating species influxes resulting from extrinsic dynamics (e.g. faunal interchanges) taking place at larger scales^2,32^.

Our model comparison strongly suggest that intrinsic diversification and migration rates (*r*_0_) also changed over the analysis interval (95% of support)^33^. The main factors driving rate shifts are ecological (functional disparity and functional saturation) and environmental (vegetation change and temperature) (Fig. 3C). To ease the comparison of diversification trends across models, we computed diversity growth (DG) in each bin as the model-averaged diversity in that bin divided by the model-averaged diversity in the previous bin^8^ (Fig. 3E; see SI Methods).

The role of ecological niche saturation (FS) on diversity-dependence processes over large timescales is still a subject of debate^6,13,30,31^. In support of the notion that increased saturation and competition can decelerate diversification, our results show that FS has a negative effect on the rate at which the system’s diversity increases (this signal is stronger in TRiPS-derived models, where FS is the first modulating factor of *r*_0_; Fig. 3E). Less crowded functional space (low FS) substantially increases rates of diversity growth compared to the strict DD models (reddish dots in Fig. 3E). As functional saturation ramps up (high FS), diversity growth plummets, with most of growth rates being similar to those expected under the pure DD scenario (Fig. 3E). Only very functionally saturated faunas show faster diversity decreases than the DD models. Overall, these findings suggest that, even at the scale of small regions, niche saturation is still an important modulator of the strength of diversity dependence. They also show that our FS estimates are truly independent from sampling and raw diversity counts (see methods), providing a single best-fit model that improves the best pure DD model (in analyses based on both TRiPS and SQS; see Dataset S1). However, in SQS- based analyses, FS-predicted diversity depletions (values of diversity growth below 1) fall mainly within the values obtained from pure DD models, which would explain why the effect of FS on the strength of the density-dependence is secondary in this subset of models (a total support of 12%). Independently of the method used to infer paleodiversity, low ecological saturation seems to facilitate species accumulation.

Shifts in canopy cover (δ^13^C) influenced Iberian Neogene diversification and migration rates, according to models based on both TRiPS and SQS (Fig. 3C and Dataset S1). Besides increasing carrying capacity, patchy, heterogeneous environments (intermediate δ^13^C values) would have slightly accelerated rates of diversity gain in comparison to pure DD models (Figs S8 and S9). In times when open habitats were more widespread in Iberian ecosystems (higher δ^13^C), the diversity growth was substantially below that predicted by pure DD models. This is in agreement with very warm periods, associated with a severe evaporation of water bodies and higher δ^18^O values^34^, showing a negative effect on the diversity growth of the system when compared to pure DD models (Fig. 2E). Environmental shifts towards more open, arid —likely less productive^35^— habitats may have increased the crowding effect of ecosystems^6^, thus hampering diversity growth by means of local speciation or migration.

We find contrasting evidence regarding the relevance of functional disparity (FD) in the Iberian mammalian faunas. Whereas the diversity curve recovered by TRiPS does not provide evidence of a controlling role of FD on the strength of diversity dependence, in models developed with the diversity trend from the SQS method FD is the second most relevant factor affecting diversification and migration rates (*r*; see Fig. 3C). If the true diversity of Iberian mammals between 15 and 2 Ma resembles the SQS estimates, our models prove that diversity in regional pools with low FD decreases faster than expected under pure diversity-dependence (Fig. 3G). As FD increases, diversity growth is faster than predicted from strict diversity-dependence. Increasing functional disparity perhaps allows the system to absorb species at higher rates by means of new ecological interactions^36^ that reduced the strength of the biotic competition. When FD is very high, the system shows both fastest faunal depletions and accelerated diversity growth than pure DD scenarios (Fig. 3G). A functionally disparate species pool could absorb species faster in systems with a high carrying capacity or if the functional space is not completely saturated (see below). In some cases, high FD can insure ecosystems functioning, buffering diversity losses during environmental fluctuations^37^. But our results show that, in other cases (e.g. under environmental disturbances^26^), functionally complex systems in a shrinking phase could be more unstable and more prone to extinction cascades. This suggests that the effect of high functional disparity over diversity may be in turn correlated with other factors.

Our modelling procedure demonstrates that, at sub-continental scales, abiotic factors have a higher influence than biotic factors (44% and 3% of total support, respectively, using the TRiPS diversity for the analyses; Fig. 2B). Nevertheless, sub-continental diversity is shaped by an interplay between both biotic and abiotic modulators (total support of 53%; Fig. 2B). At the spatial and temporal scale of our analyses, the relevance of competition and niche saturation dynamics, which are expected to rule species richness in communities^6,31^ or small regions over short timescales^7^, becomes faded and abiotic drivers start taking over^4^.

We also see evidence that terrestrial species richness in sub-continental regions is the outcome of an intricate interconnection of multi-scale processes. Models that integrate local- and broad-scale factors have 80% of total support (against 17% and 3% of the total support aggregated by models based exclusively on local- and large-scale drivers, respectively; Fig. 2C). Future studies investigating the drivers of regional diversity trends should be conducted following a multi-scalar perspective and should avoid relying exclusively on a single factor. Importantly, although global temperature^38^ may be a good predictor of diversification at global scale^8,39^, it does not seem to be a direct modulator of diversity patterns at the scale of small regions, even over geological temporal scales (total support of 3%), which highlights its unsuitability for sub-continental macroevolutionary studies^16^. Even if significant correlations are found between global temperature and local diversity, this should be interpreted, not as a direct cause-effect, but as a complex interrelationship with other intermediate factors (e.g. biogeographic events, environmental heterogeneity, etc.)^40,41^.Our findings portray sub-continental species pools as complex and dynamic systems where diversity is perpetually imbalanced and at the mercy of the interplay between local and broad-scale factors. Competition and ecological dynamics still play relevant roles at these spatial scales, but physical and environmental drivers, such us tectonics triggering intercontinental faunal dispersals, are the major contributors to sub-continental diversity.

## Methods

The Iberian Peninsula stands out for containing the highest density of Neogene mammal localities of the European continent, and its fossil record has been the object of taxonomic study for more than six decades^42^. The localities in our dataset have been precisely dated using a maximum likelihood approach^33^, span from 15 to 2 Ma, and include 721 occurrences of 209 species. We estimate paleodiversity curves using two different methods: a maximum likelihood method (True Richness estimated using a Poisson Sampling, or TRiPS^23^), and a subsampling method (the Shareholder Quorum Subsampling method, or SQS^24^) (see supplementary methods and Fig. S1).

We then use equations in Ezard and Purvis^8^ to model 13 million years of terrestrial mammal diversity. We build diversity models that assume different diversity-dependence (DD) scenarios. In these DD models the diversification + migration rate (*r*) is a function of diversity in relation to a diversity cap (*K*) and the diversification + migration rate when diversity is 0 (the intrinsic or unconstrained diversification + migration rate, *r*_*0*_). The “contest” model reflects a logistic diversity accumulation where diversity plateaus around a diversity limit (*K*)^5,6^. In the “expansion and collapse” model, the system follows strong species depletions (negative *r*) once *K* is surpassed^8^. In the “damped increase” scenario the *crowding effect* is weak, and *r* is just slightly affected by diversity^8^. We can use these three models (here called pure DD models), where *K* is fixed, as a basis, and then incorporate into the equations factors that modulate *K* and/or the intrinsic diversification + migration rate (*r*_*0*_). We also evaluate models where *r0* and *K* track these factors but without a diversity-dependence effect.

A total of seven factors are added to the models: functional disparity (FD), functional saturation (FS) —both obtained from body size, diet and locomotion data—, type of vegetation (based on δ^13^C isotopic values), temperature/aridity (as reflected by δ^18^O isotopic records), global climate^38^, and biogeographic affinities with the region comprised by Europe and the Middle East (EME) and Africa (which are estimated as the average faunal similarity with over 700 localities in EME and Africa through time). From the combination of these seven factors and the different types of models (either *K*, or *r*_*0*_, or both are a function of one of these factors), we run a total of 206 models for each of the two diversity curves (obtained using TRiPS and SQS). Models are compared using their AICc weights^8^, and the overall contribution of different factors is assessed aggregating AICc weights across models (see Dataset S1). We run all the analyses using R^43^ code available from Ezard and Purvis^8^. Further methodological details are available in the SI Methods.

## Electronic supplementary material

Supplementary Info

Dataset 1
