# Supplementary material for: Multi-scale interplays of biotic and abiotic drivers shape mammalian sub-continental diversity over millions of years
Source: Sci Rep. 2018 Sep 7;8:13413. doi: 10.1038/s41598-018-31699-6 (PMC6128930; doi:10.1038/s41598-018-31699-6)
Supplement: Supplementary file 1 — Supplementary Info [file 41598_2018_31699_MOESM1_ESM.pdf]

Multi-scale interplays of biotic and abiotic drivers shape mammalian sub-continental diversity over millions of years

Juan L. Cantalapiedra [1,2], M. Soledad Domingo [3,4], Laura Domingo[4,5,6]

e-mails: jlopezcant@gmail.com, sdomingo@ebd.csic.es, lauradomingo@geo.ucm.es

[1] Museum für Naturkunde, Leibniz-Institut für Evolutions und Biodiversitätsforschung, Invalidenstraße 43, 10115 Berlin, Deutschland.

[2] Dpto Ciencias de la Vida, Universidad de Alcalá, 28805 Alcalá de Henares, Madrid, Spain.

[3] Dpto Ecología Evolutiva, Estación Biológica de Doñana (CSIC), Américo Vespucio 26, 41092 Sevilla, Spain.

[4] Dpto Paleontología, Facultad de Ciencias Geológicas, Universidad Complutense de Madrid, José Antonio Nováis 2, 28040 Madrid, Spain.

[5] Dpto Geología Sedimentaria y Cambio Medioambiental, Instituto de Geociencias (UCM,CSIC), C/ José Antonio Nováis 12, 28040 Madrid, Spain.

[6] Earth and Planetary Sciences Department, University of California SantaCruz, 1156 HighStreet, CA 95064, USA.

SI Methods

Fossil data. Our fossil dataset includes 94 Iberian fossil sites with macro-mammals remains whose ages span from 16 Ma to around 2 Ma. We excluded from the database fossil sites from the Northeastern Iberian Peninsula (i.e., Vallès-Penedès and Seu d'Urgell Basins) since they have been traditionally assigned to a different bioprovince[1], and, also, because we do not have isotopic data from these basins (see below). Seventy seven of the fossil sites included in this study had been precisely dated using the Maximum Likelihood Appearance Event Ordination (ML-AEO)[2]. The other 17 localities are assigned to local mammal age units (MN units; see temporal ranges assigned to each locality in Dataset S1). After discarding fossil occurrences that were not identified at the species level, our dataset includes 721 occurrences of 209 species.

Taxonomic diversity through time. Estimates of past diversity that use the fossil record should account for sampling bias. Our approach uses two different methods of paleodiversity estimation: the True Richness estimated using a Poisson Sampling model (TRiPS)[3], and the Shareholder Quorum Subsampling method (SQS)[4]. Taking advantage of the precise calibration of the fossil sites in our dataset, we performed a sliding time window analyses to avoid edge effects. We sampled occurrence data within a time window of 1 myr every 0.25 myr. Occurrence data from the 17 localities assigned to MN units were assigned a random age within the MN unit temporal range prior to the analyses.

The TRiPS approach jointly models biodiversity and the sampling process within each time bin, here each 1 myr window. The method provides maximum likelihood estimates of both sampling rates and species richness[3]. We retain the maximum likelihood estimate of biodiversity for subsequent analyses.

The SQS is a subsampling approach. The SQS is based on each species 'coverage', the percentage of the total occurrences belonging to that species in each focus interval. This 'coverage' is modified by multiplying by Good's u (the proportion of occurrences of non-singleton data, which is a proxy for sampling completeness; Fig. S1E) to estimate the real frequency[4]. Good's u also marks the maximum quorum level (q) to which we can subsample (fewer intervals can be subsampled as we increase this *quorum*) (Fig. S1E). We estimate Good's u for the 100 resampled versions of the dataset, and explore the percentage of time windows where at least 10 from 100 of the Good's u values fell above 0.4 and 0.5, since a q below 0.4 is not recommended[4]. A q of 0.4 still retains information for 84% of the time windows, whereas using 0.5 drops a 25% of the data (Fig. S1E). Thus, we use a q of 0.4 for estimating SQS diversity through time.

We estimate TRiPS and SQS diversity for the 100 resampled versions of the dataset. For TRiPS, we exclude estimates associated with a sampling probability below 0.25 (Fig. S1C), because these yield non-realistic diversity values and very broad confidence intervals. Also, time windows for which less than 10 richness estimates are computed across the 100 resampled datasets are also discarded. For SQS estimates, we retain values from time windows where at least 10 estimates

were obtained (10 estimates are above the quorum of 0.4 across resampled versions of the data). To synthesize the obtained clouds of points from the resampled TRiPS and SQS estimates (Fig. S1), we fit locally weighted (LOESS) curves to the data (Fig. S1D and S1F). An optimal smoothing parameter is selected using an Akaike Information Criterion (AIC)[5] so that the resulting curves capture the general trend, and reduces influence of both extreme points and the noise caused by the temporal uncertainty of in our data. Also, this kind of approach allows to predict values in short intervals bins where we have fewer direct observations (e.g. intervals where Good's *u* is below the quorum value of 0.4, for example between 5.5 and 4.75 Ma; see Fig. S1F). The resulting curves are retained for posterior analyses. LOESS curves were also used for the rest of the time series (Fig. 1 and methods below).

Dynamics of functional space through time. We use three categorical traits to describe the functional strategy of each species: body size, diet and locomotion. Categorical traits are highly informative[6] and allow to include more fossil taxa into the analyses. For body size we followed the classification of Andrews[7]: < 1 kg, 1-10 kg, 10-45 kg, 45-90 kg, 90-180 kg, 180-360 kg, 360-1000 kg, and > 1000 kg. We use the following diet categories: grazer, mixed-feeder grazer, mixed feeder, opportunistic feeder, browser mixed feeder, browser, plant dominated omnivore, omnivore, animal dominated omnivore, insectivore, carnivore-invertebrates, molluscivore, carnivore, meat bone, and hypercarnivore. Species are assigned to one of these locomotion modes: ambulatory, brachiation, cursorial, excavator, graviportal, knuckle walking, mediportal, scansorial, and semi-aquatic. We gather functional information from the literature[8-24], isotopic signal, and the New and Old Worlds database[25] for 205 species. We only incorporate into the analyses species with information for at least two traits. 196 species are retained (~ 94% of the total fossil diversity) with a total of 77 observed combinations of functional traits (functional entities). Body size and dietary traits are treated as ordered categorical variables (same ordination as shown above) in the analyses. See a list of the 209 species with available functional information in Dataset S1.

The functional traits are used to investigate ecological functionality of the regional species pool through time. We compute overall functional space by calculating Gower's distances[26] applying the function *daisy* in the R package *cluster* to our three traits. Gower's metric allows the inclusion of missing data (here only 6.6%) since the method rescales distances based on the amount of information available[26]. We estimate functional disparity (FD) and functional saturation (FS)[27]. FD is measured as the mean distances among the taxa in a given time window. FS is calculated as the negative of the mean nearest-neighbour distance (NND) among the taxa (saturation increases when NNDs are smaller). FD reflects the overall functional diversity, whereas FS measures the relative saturation of taxa in the functional space[28] (functional partitioning). 1 myr time windows are placed every 0.25 myr through the study interval and we extract the species with functional information in each window. FD and FS are only computed for time windows that contain at least 4 localities with species with functional information for two of the traits, resulting in a range of 8 to 41

species across bins. We find no significant correlation between FD and the number of species with functional information sampled in each window (Kendall's $\tau = 0.157$; $P = 0.118$). This suggests that the trends observed are the result of true biological signal, and not just an artefact of paleontological sampling. FS could also be sensitive to sampling[28]. Poor sampled windows are expected to show less extreme nearest-taxon distances (a lower range of NNDs)[29]. Our data show the opposite trend, with the standard deviation of the observed NNDs decreasing with species richness ($\tau = -0.463$; $P < 0.001$), indicating that the signal of the less rich temporal bins is not affected by sampling. FD and FS are scaled from 0 to 1 to ease model fitting (see below) and graphical representation (Fig. 1B). LOESS curves are applied to the estimated values of FD and FS through time (Fig. S3), using an Akaike Information Criterion (AIC) for selecting the appropriate smoothing parameter[5]. These curves are retained for posterior analyses.

Biogeography. Large scale biogeographic events (e.g. intercontinental faunal interchanges) should influence terrestrial communities composition[30-32]. To test whether such episodes also impact diversity trends in small regions, we investigate the influence of European and African faunas on our species pool through time. We estimate the mean Raup-Crick similarity of the Iberian localities with those from Europe and Middle East, and Africa. Presence-absence data for all the localities was obtained from the NOW database[25] and the analyses were carried out at the genus level. Only localities with at least 5 genera were considered.

Isotopic data. Sampled specimens for stable isotope analyses belong to eight mammalian families (Elephantidae, Gomphoteriidae, Equidae, Rhinocerotidae, Bovidae, Cervidae, Suidae and Giraffidae) from 45 Iberian fossil sites (Fig. S5). We compile tooth enamel isotopic data from publications[33-35]. We complete our dataset with new data from 4 Iberian localities (Carpetana, Relea, Lugarejo and Corral de Lobato), as well as with unpublished *Hipparion* stable isotope data from Nombrevilla 1, Masía del Barbo 2B, Vivero de Pinos, Puente Minero, Masada del Valle, Los Mansuetos, Arquillo 1, Las Casiones, Milagros, La Calera, Orrios 1 and Villalba Alta.

The teeth used in this study are archived at the Museo Nacional de Ciencias Naturales-CSIC (Madrid, Spain), Museo Geominero-Instituto Geológico y Minero de España (Madrid, Spain), and Fundación Conjunto Paleontológico de Teruel-Dinópolis (Teruel, Spain). The carbon and oxygen isotope results are reported in the δ-notation $\delta^{H}X_{sample}=[(R_{sample}-R_{standard})/R_{standard}]\times1000$, where X is the element, H is the mass of the rare, heavy isotope, and $R={}^{13}C/{}^{12}C$ or ${}^{18}O/{}^{16}O$. Vienna Pee Dee Belemnite (VPDB) is the standard for $\delta^{13}C$ values and $\delta^{18}O$ values for both apatite carbonate and phosphate are reported relative to Vienna Standard Mean Ocean Water (VSMOW).

Tooth enamel samples (n=728) were analyzed for the carbon and oxygen isotope composition of carbonate in bioapatite ($\delta^{13}C$ and $\delta^{18}O_{CO3}$, respectively). Chemical pre-treatment followed the one described in Domingo et al.[33]. Isotopic analyses were conducted at the stable isotope laboratories of the University of California Santa Cruz using a ThermoScientific MAT253 dual inlet isotope ratio mass spectrometer coupled to a ThermoScientific Kiel IV carbonate device and of the University of

Minnesota using a ThermoScientific MAT252 dual inlet isotope ratio mass spectrometer coupled to a ThermoScientific Kiel II carbonate device.

The $\delta^{18}O$ values of phosphate in bioapatite ($\delta^{18}O_{PO4}$) were measured on 372 tooth enamel samples. The chemical treatment is described in Domingo et al.[33]. Analyses were performed at the stable isotope laboratories of the University of California Santa Cruz using a ThermoFinnigan Delta plus XP IRMS coupled to a ThermoFinnigan High Temperature Conversion Elemental Analyzer (TCEA) and of the University of Kansas using a Thermo Finnigan MAT 253 IRMS coupled to a ThermoFinnigan TCEA.

Among mammalian herbivores, there is a contrast between obligate drinkers (animals that obtain most of their water from drinking) and non-obligate drinkers (those obtaining water mainly from plant water and metabolic water) [36]. The first group drinks water daily or consumes non-leafy parts of plants (which contain water that has not experienced evaporation). As a result, these animals record more faithfully local meteoric water $\delta^{18}O$ values as well as temperature. Non-obligate drinkers can survive with little or no drinking water. They obtain most of their water from leaf water, which is prone to strong evaporative ^{18}O-enrichment. Hence the $\delta^{18}O$ values of non-obligate drinking herbivores increase relative to meteoric water values (as monitored by obligate drinking herbivores) with increasing aridity [37]. For this reason, we use $\delta^{18}O$ values from obligate drinkers to construct our isotopic curves. A total of 622 records of $\delta^{13}C$ and 515 records of $\delta^{18}O$ are included in our analyses.

Final data-points. We used LOESS curves to extrapolate values of TRiPS and SQS diversity, FD, FS, $\delta^{13}C$, $\delta^{18}O$ and faunal similarity. We had estimated TRiPS and SQS diversity, FD, FS and biogeographic affinities for time windows every 0.25, with FD and FS estimates covering a slightly shortest time span (15 - 2 Ma). This would yield 53 time points with data. However, at each time point, our modeling approach requires the current diversity (x) and the diversity in the next time point (y)[38]. As a result, the most recent age for which we have the diversity for the next time point is 2.25, and our dataset generates a total of 52 data points for each variable, from 15 to 2.25 Ma, every 0.25 myr.

Diversity models. We used a maximum likelihood approach[38] to assess the effect of diversity-dependence (DD), functional space, biogeography and environment in diversification+migration rates and diversity limits. The basis of this approach are three basic models of competition (see detailed model descriptions, formulas and R code in Ezard and Purvis)[38]. These basic models assume constant carrying capacity (K) and constant intrinsic diversification+migration rate (the rate when diversity tends to zero or unconstrained rate, r_0). We term this basic models ‘pure DD models’. The realized diversification+migration rate (r) is a function of diversity in relation to K and r_0. The ‘bounded contest’ model is the analog of logistic growth. In this model diversity plateaus (r becomes 0) as it reaches the carrying capacity (K). In the ‘expansion and collapse’ scenario, adding species to the regional species pool produces the contraction of the niche breath of the

other species. As a result, there is a diversity limit where competition is analogous to the 'bounded contest', but beyond this point the system is not sustainable any longer and diversity collapses (*r* becomes negative). In the 'damped increase', the intensity of competition is included as the competition coefficient (*c*). Values of $c \sim 1$ would mimic the 'bounded contest'. For $c > 1$, the model would work similar to an 'expansion and crash' scenario. For $c < 1$, diversity would never stop diversification completely. In particular, as *c* tends to zero, *K* tends to infinite, and thus diversity would only depend on diversification (a true unbounded scenario).

More complex versions of these DD models can be built by incorporating factors (e.g. FD, FS, $\delta^{13}C$, $\delta^{18}O$, etc.) that modulate *K* and r_0 over time[38]. We also build models where *K* and r_0 directly depend on such factors, but with no diversity-dependence involve. In total, 412 models were fitted (206 based on TRiPS diversity and 206 based on SQS diversity; see descriptions of the models in Dataset S1). We used AICc weights to evaluate the statistical support of each model. Akaike weights measure the relative probability of each model being the best one among those compared. Specifically, we compute aggregated AICc weights across different factors to investigate their contribution to the fit of the models (Dataset S1). To test that the observed pattern is not a result of the amalgamation of models with poor support, we also estimate AICc weights from the best performing model (lowest AICc) in each factor's category (Dataset S1).

To interpret the contribution of each factor to the models, relevant parameters are model-averaged by multiplying them by the AICc weight of the respective model and then summing the results across all models[38]. We compare averaged parameters from the pure DD models with those where a given factor modulated *K* or r_0. In order to assess how a given factor impacts *K*, we look at the differences in AICc-averaged diversity between pure DD models and models where *K* was a function of that factor and plot these differences against values of the factor (Fig. 3, S8 and S9). To assess how a given factor impacts r_0, we look at the differences in AICc-averaged diversity between pure DD models and models where r_0 was function of that factor and plot these differences against values of the factor (Fig. 3, S8 and S9).

References

1 Gómez Cano, A. R., Hernández Fernández, M. & Álvarez-Sierra, M. A. Biogeographic provincialism in rodent faunas from the Iberoccitanian Region (southwestern Europe) generates severe diachrony within the Mammalian Neogene (MN) biochronologic scale during the Late Miocene. *Palaeogeogr. Palaeoclimatol. Palaeoecol.* **307**, 193-204, doi:10.1016/j.palaeo.2011.05.014 (2011).

2 Domingo, M. S., Badgley, C., Azanza, B., DeMiguel, D. & Alberdi, M. Diversification of mammals from the Miocene of Spain. *Paleobiology* **40**, 196-220 (2014).

3 Starrfelt, J. & Liow, L. H. How many dinosaur species were there? Fossil bias and true richness estimated using a Poisson sampling model. *Philos. Trans. R. Soc. Lond. B Biol. Sci.* **371**, 20150219-20150210, doi:10.1098/rstb.2015.0219 (2016).

4 Alroy, J. in *Quantitative Methods in Paleobiology: Paleontological Society Papers* Vol. 16 (eds J. Alroy & G. Hunt) 55-80 (2010).

5 Hurvich, C. M., Simonoff, J. S. & Tsai, C. L. Smoothing parameter selection in nonparametric regression using an improved Akaike Information Criterion. *J. R. Stat. Soc. B* **60**, 271–293 (1998).

6 Fortelius, M. *et al.* An ecometric analysis of the fossil mammal record of the Turkana Basin. *Philos. Trans. R. Soc. Lond. B Biol. Sci.* **371**, 20150232, doi:10.1098/rstb.2015.0232 (2016).

7 Andrews, P., Lord, J. M. & Evans, J. P. Patterns of ecological diversity in fossil and modern mammalian faunas. *Biol. J. Linn. Soc.* **11**, 177-205, doi:10.1111/j.1095-8312.1979. tb00034 (1979).

8 Gromolard, C. & Guerin, C. Mise au point sur*Parabos cordieri*(de Christol), un bovidé (Mammalia, Artiodactyla) du Pliocène d' Europe occidentale. *Geobios* **13**, 741–755, doi:10.1016/S0016-6995(80)80053-2 (1980).

9 Janis, C. Correlation of Cranial and Dental Variables with Dietary Preferences in Mammals: A Comparison of Macropodoids and Ungulates. *Mem. Queensl. Mus.* **28**, 349-366 (1990).

10 Alberdi, M. T., Prado, J. L. & Ortiz-Jaurequizar, E. Patterns of body size changes in fossil and living Equini (Perissodactyla). *Biol. J. Linn. Soc.* **54**, 349-370, doi:10.1111/j.1095-8312.1995.tb01042.x (1995).

11 Alberdi, M. T., Prado, J. L. & Ortiz-Jaurequizar, E. A quantitative review of European stenonoid horses. *J. Paleontol.* **72**, 371-387, doi:http://dx.doi.org/10.1017/S0022336000036350 (1998).

12 Sánchez, I. M., Salesa, M. J. & Morales, J. Revisión sistemática del género Anchitherium Meyer 1834 (Equidae; Perissodactyla) en España. *Estud. Geol.* **54**, 39-63, doi:10.3989/egeol.98541-2204 (1998).

13 Cerdeño, E. & Sánchez, B. Intraspecific variation and evolutionary trends of*Alicornops simorrense*(Rhinocerotidae) in Spain. *Zool. Scr.* **29**, 275-305 (2000).

14 Agustí, J. & Antón, M. *Mammoths, Sabertooths, and Hominids.* (Columbia University Press, 2002).

15 Ortiz-Jaurequizar, E. & Alberdi, M. El patrón de cambios en la masa corporal de los Hipparionini (Perissodactyla, Equidae) de la península ibérica durante el Mioceno Superior-Plioceno Superior. *Coloquios de Paleontología* **1**, 499-509 (2003).

16 Prado, J. L., Alberdi, M. T., Azanza, B. & Rodríguez, J. in *Miscelánea en homenaje a Emiliano Aguirre. Paleontología* (eds E. Baquedano & S. Rubio) 464-479 (Museo Arqueológico Regional, Alcalá de Henares, 2004).

17 Kaiser, T. M. & Rossner, G. E. Dietary resource partitioning in ruminant communities of Miocene wetland and karst palaeoenvironments in Southern Germany. *Palaeogeogr. Palaeoclimatol. Palaeoecol.* **252**, 424-439, doi:10.1016/j.palaeo.2007.04.013 (2007).

18 Arribas, A. & Garrido, G. in *Cuadernos del Museo Geominero, no 10. Instituto Geológico y Minero de España, Madrid,* Vol. 10 (ed A. Arribas) 337-364 (Instituto Geológico y Minero de España, 2008).

19 Bibi, F. & Güleç, E. S. Bovidae (Mammalia: Artiodactyla) from the late Miocene of Sivas, Turkey. *J. Vertebr. Paleontol.* **28**, 501-519, doi:10.1671/0272-4634(2008)28[501:BMAFTL]2.0.CO;2 (2008).

20 DeMiguel, D., Fortelius, M., Azanza, B. & Morales, J. Ancestral feeding state of ruminants reconsidered: earliest grazing adaptation claims a mixed condition for Cervidae. *BMC Evol. Biol.* **8**, doi:10.1186/1471-2148-8-13 (2008).

21 Perales, R., Serrano, H., García Yelo, B. A. & Hernández Fernández, M. Palaeoenviromental inferences of the Middle Miocene from Somosaguas (Pozuelo de Alarcón, Madrid) based on the corporal sizes structure its mammal fauna. *Paleolusitana* **1**, 317-325 (2009).

22 Tseng, Z. J. Cranial function in a late Miocene Dinocrocuta gigantea (Mammalia: Carnivora) revealed by comparative finite element analysis. *Biol. J. Linn. Soc.* **96**, 51-67, doi:10.1111/j.1095-8312.2008.01095.x (2009).

23 Alba, D. M. *et al.* Middle Miocene tragulid remains from Abocador de Can Mata: The earliest record of Dorcatherium naui from Western Europe. *Geobios* **44**, 135-150, doi:10.1016/j.geobios.2010.10.003 (2011).

24 Cantalapiedra, J. L., Prado, J. L., Hernández Fernández, M. & Alberdi, M. T. Decoupled ecomorphological evolution and diversification in Neogene-Quaternary horses. *Science* **355**, 627–630, doi:10.1126/science.aag1772 (2017).

25 Fortelius, M. New and Old Worlds Database of Fossil Mammals (NOW). University of Helsinki. http://www.helsinki.fi/science/now/. (2016).

26 Lloyd, G. T. Estimating morphological diversity and tempo with discrete character-taxon matrices: implementation, challenges, progress, and future directions. *Biol. J. Linn. Soc.* **118**, 131-151, doi:10.5061/dryad.gp16s (2016).

27 Foote, M. Nearest-Neighbor Analysis of Trilobite Morphospace. *Syst. Zool.* **39**, 371-382, doi:10.2307/2992357?ref=search-gateway:e8c6a3c306372d9addc2e2a559b3c097 (1990).

28 Bapst, D. W., Bullock, P. C., Melchin, M. J., Sheets, H. D. & Mitchell, C. E. Graptoloid diversity and disparity became decoupled during the Ordovician mass extinction. *Proc. Natl. Acad. Sci. U. S. A.* **109**, 3428-3433, doi:10.1073/pnas.1113870109 (2012).

29 Schum, M. Phenetic Structure and Species Richness in North and Central American Bat Faunas. *Ecology* **65**, 1315-1324, doi:10.2307/1938336?ref=search-gateway:d2d9bc77691f522a4a3a950454a86b04 (1984).

30 Vrba, E. S. Mammals as a key to evolutionary theory. *J. Mammal.* **73**, 1-28, doi:10.2307/1381862 (1992).

31 Pickford, M. & Morales, J. Biostratigraphy and palaeobiogeography of East Africa and the Iberian Peninsula. *Palaeogeogr. Palaeoclimatol. Palaeoecol.* **112**, 297-322, doi:10.1016/0031-0182(94)90078-7 (1994).

32 Bacon, C. D. *et al.* Biological evidence supports an early and complex emergence of the Isthmus of Panama. *Proc. Natl. Acad. Sci. U. S. A.* **112**, 6110-6115, doi:10.1073/pnas.1423853112 (2015).

33 Domingo, L., Cuevas-González, J., Grimes, S. T., Hernández Fernández, M. & Lopez-Martinez, N. Multiproxy reconstruction of the palaeoclimate and palaeoenvironment of the Middle Miocene Somosaguas site (Madrid, Spain) using herbivore dental enamel. *Palaeogeogr. Palaeoclimatol. Palaeoecol.* **272**, 53-68, doi:10.1016/j.palaeo.2008.11.006 (2009).

34 Domingo, L., Koch, P. L., Grimes, S. T., Morales, J. & López-Martínez, N. Isotopic paleoecology of mammals and the Middle Miocene Cooling event in the Madrid Basin (Spain). *Palaeogeogr. Palaeoclimatol. Palaeoecol.* **339-341**, 98-113, doi:10.1016/j.palaeo.2012.04.026 (2012).

35 Domingo, L. *et al.* Late Neogene and Early Quaternary Paleoenvironmental and Paleoclimatic Conditions in Southwestern Europe: Isotopic Analyses on Mammalian Taxa. *PLoS ONE* **8**, e63739, doi:10.1371/journal.pone.0063739.s004 (2013).

36 Kohn, M. J. Predicting animal $\delta^{18}O$: accounting for diet and physiological adaptation. *Geochim. Cosmochim. Ac.* **60**, 4811–4829, doi:10.1016/S0016-7037(96)00240-2 (1996).

37 Levin, N. E., Cerling, T. E., Passey, B. H., Harris, J. M. & Ehleringer, J. R. A stable isotope aridity index for terrestrial environments. *Proc. Natl. Acad. Sci. U. S. A.* **103**, 11201–11205, doi:10.1073/pnas.0604719103 (2006).

38 Ezard, T. H. G. & Purvis, A. Environmental changes define ecological limits to species richness and reveal the mode of macroevolutionary competition. *Ecol. Lett.* **18**, 899–906, doi:10.1111/ele.12626 (2016).

Supporting Figures and Dataset Captions

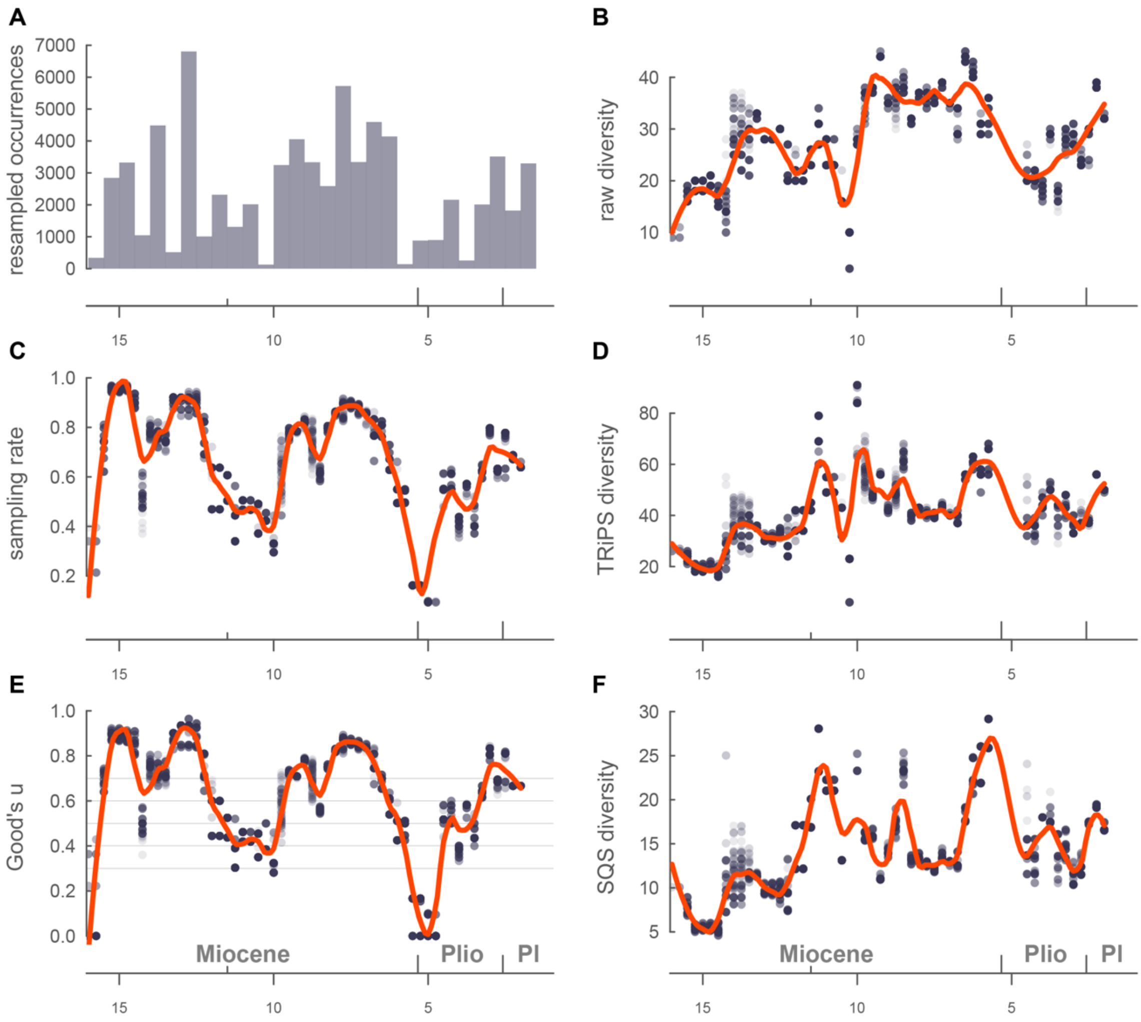

Figure S1. A histogram of the ages of the 721 occurrences of our dataset resampled 100 times (72100 values) shows the quality of the Iberian fossil record through time (A). Based on this data, our sliding window approach (see methods) yields the raw biodiversity shown in B. C, sampling probability as estimated from TRiPS. Only estimates associated with a sampling rate above 0.25 were retained (see methods). D, maximum likelihood estimates of biodiversity through time yielded by the True Richness estimated using a Poisson Sampling model (TRiPS)[3]. Good's *u* (E) reflects the proportion of occurrences of non-singleton data in each window, which is a proxy for sampling completeness. Good's *u* also marks the maximum quorum level (*q*) to which we can subsample using the Shareholder Quorum Subsampling (SQS)[4] method (fewer intervals can be subsampled as we increase this quorum). After setting a quorum of 0.4, we estimated SQS diversity (F) for the 100 resampled versions of the dataset, retaining SQS values from time windows where at least 10 SQS estimates (points) were obtained. In all plots we fitted LOESS curves applying an optimal smoothing parameter that was selected using an Akaike Information Criterion (AIC) so that the

resulting curve captures the general trend and reduces influence of both extreme points and the noise caused by the temporal uncertainty in our data. Plio, Pliocene. Pl, Pleistocene.

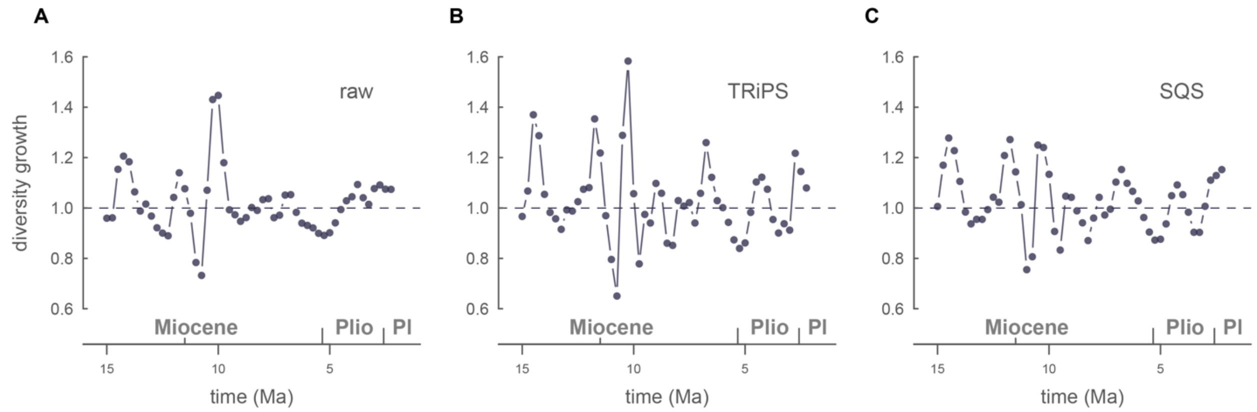

Figure S2. Diversity growth (diversity in one bin divided by the diversity in the previous bin) through time estimated from raw diversity (A), and diversity estimated using TRiPS (B) and SQS (C). See Fig. S1 for estimation of the three different diversity curves. Values below 1 reflect diversity depletions.

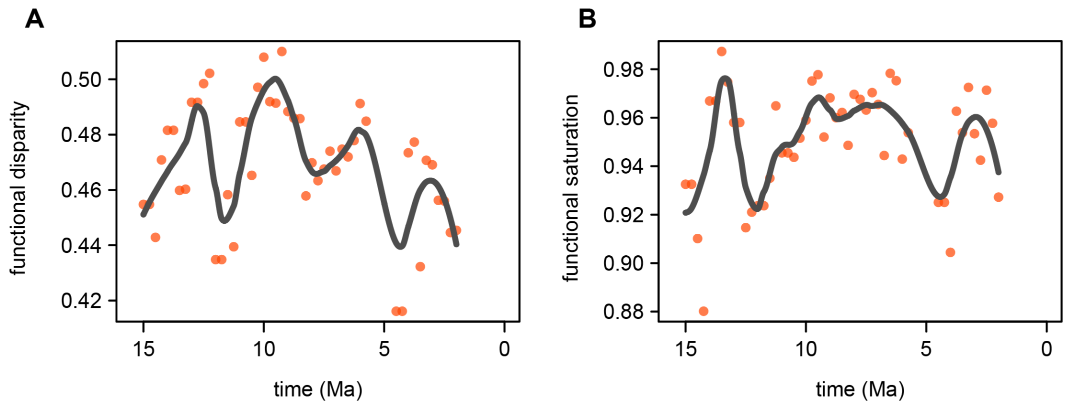

Figure S2. Within-bin estimates of functional diversity (FD) and functional saturation (FS). LOESS curves were retained for posterior analyses (see Fig. 1B).

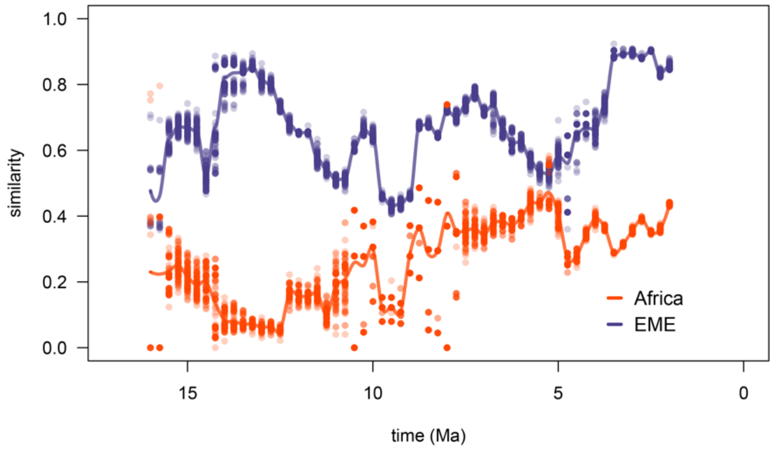

Figure S3. Similarity of Iberian large mammals faunas with Africa and Europe and the Middle East (EME). LOESS curves were retained for posterior analyses (see Fig. 1C).

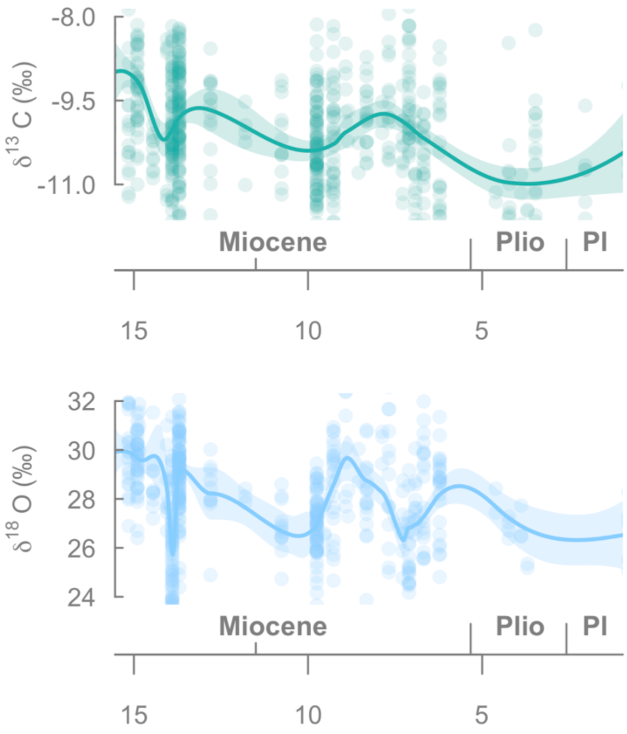

Figure S4. Stacked $\delta^{13}C$ and $\delta^{18}O$ records from Iberian herbivore fossil tooth enamel. LOESS curves were retained for posterior analyses (see Fig. 1D and 1E).

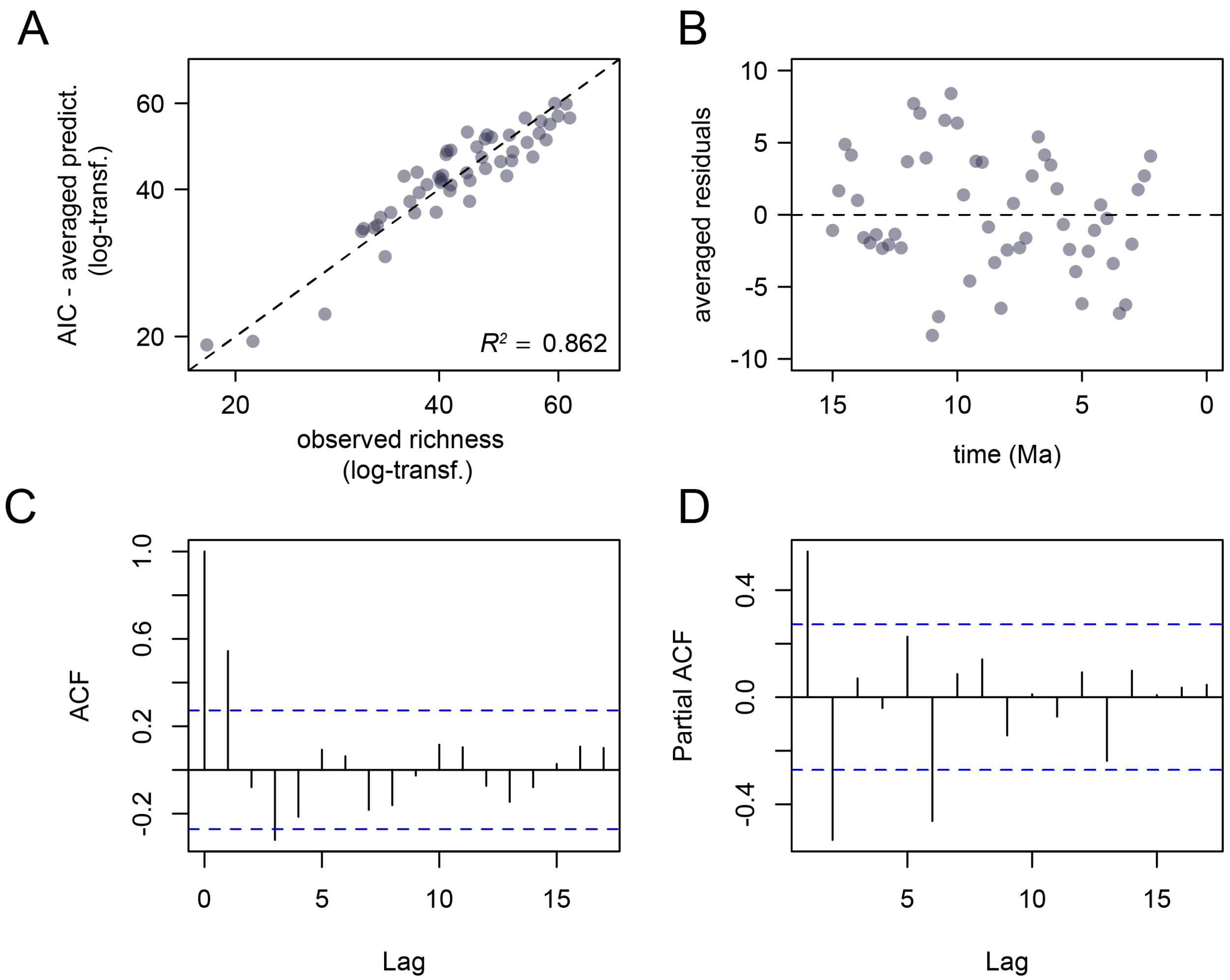

Figure S5. Our model-averaged diversity predictions explain 86% of the real variation in species richness as estimated by TRiPS (A). The dashed lines in A and B represent a perfect fit. The resulting residuals don't show a time-dependent trend (B). Both the autocorrelation (C) and the partial auto-correlation (D) functions show no sign of autocorrelation among model-averaged residuals. Orange dashed lines in C and D show a significance level of 0.05.

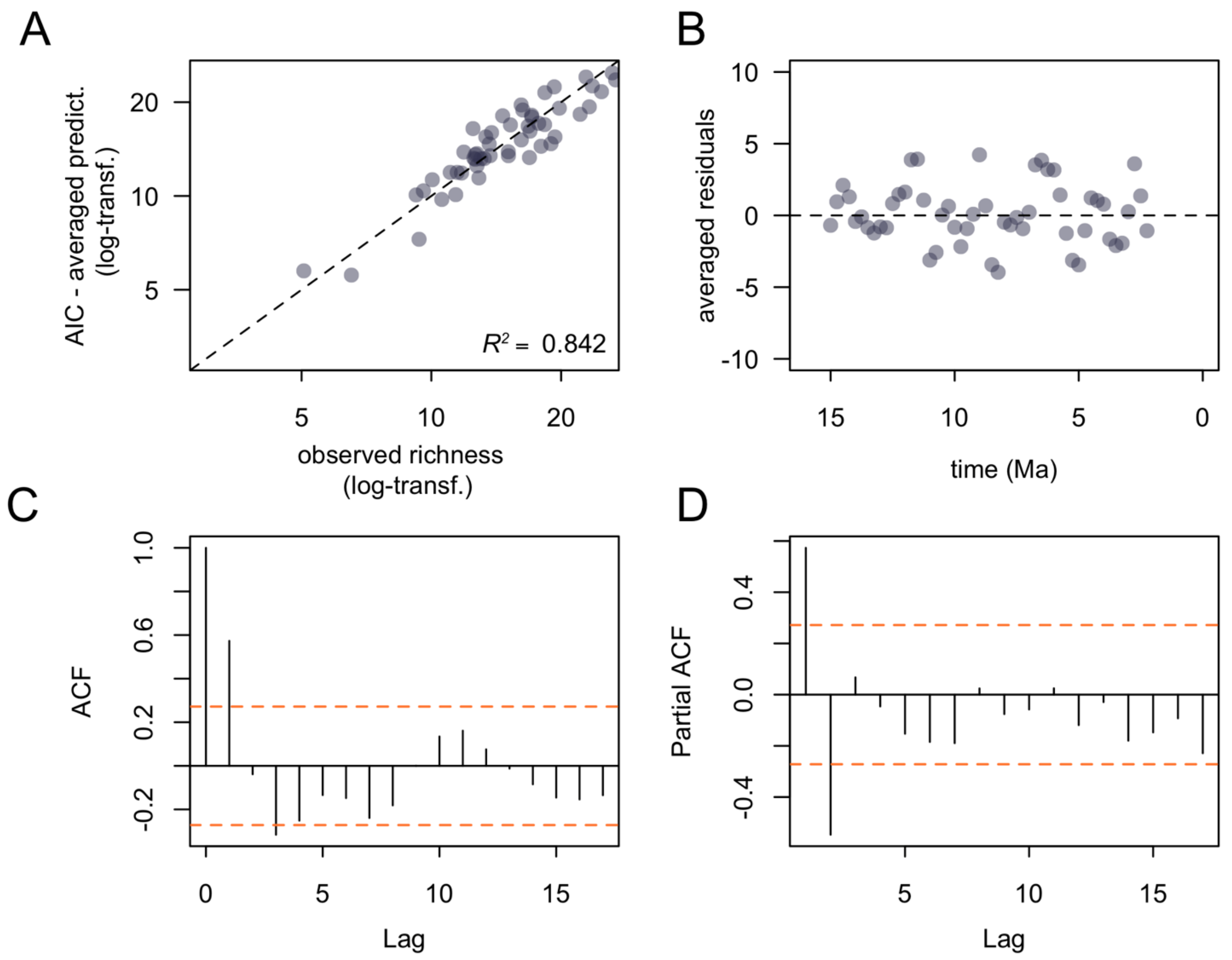

Figure S6. Our model-averaged diversity predictions explain 84% of the real variation in species richness as estimated by the SQS method (A). The dashed lines in A and B represent a perfect fit. The resulting residuals don't show a time-dependent trend (B). Both the autocorrelation (C) and the partial auto-correlation (D) functions show no sign of autocorrelation among model-averaged residuals. Orange dashed lines in C and D show a significance level of 0.05.

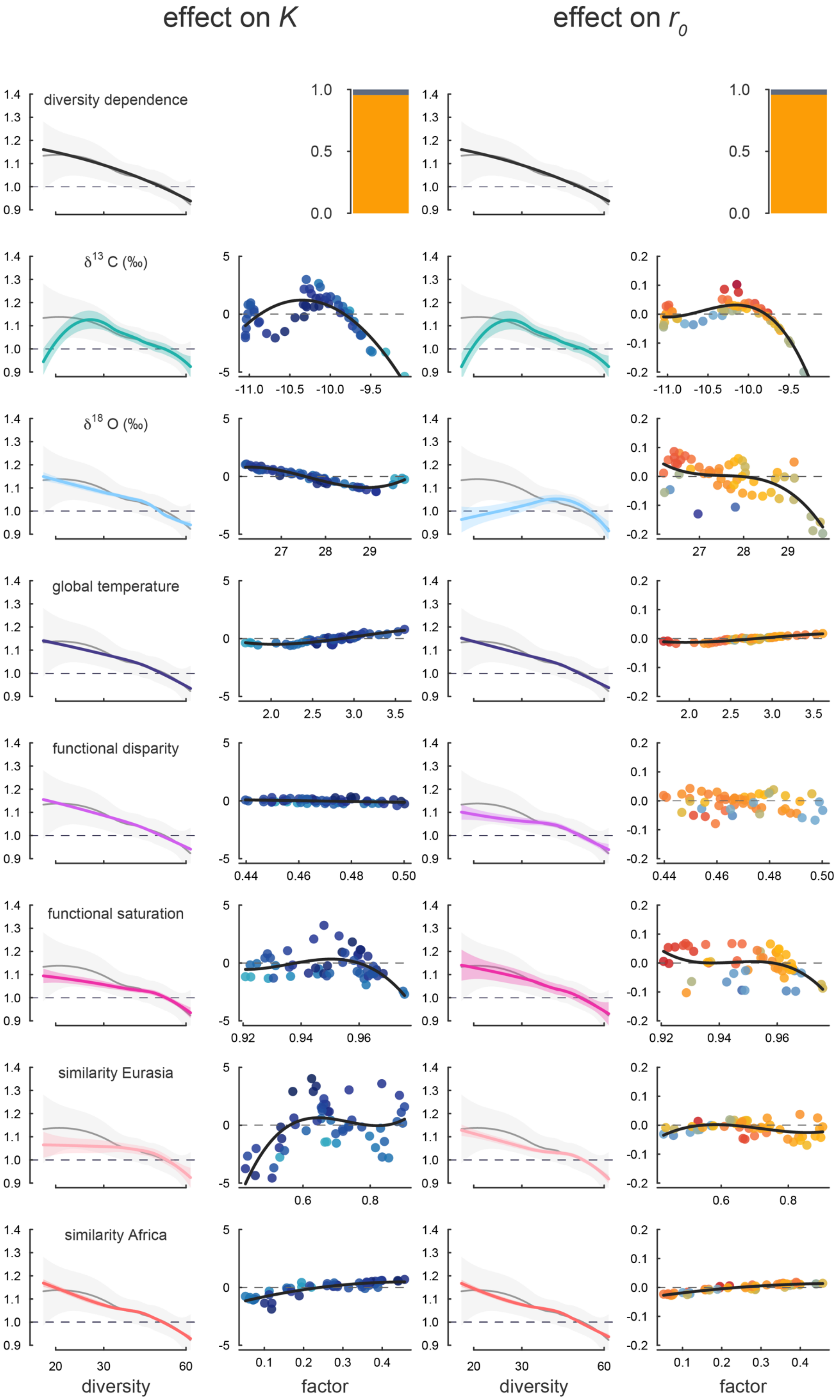

effect on K
effect on r_0
diversity dependence
δ^{13}C (‰)
δ^{18}O (‰)
global temperature
functional disparity
functional saturation
similarity Eurasia
similarity Africa
diversity
factor
diversity
factor

Figure S7. Results of model- averaging parameters from models based on TRiPS-estimated diversity. Bar-plots show the support for pure DD models in grey (fixed K and r_0, respectively). Effect of different factors on the strength of diversity-dependence. The first column shows the rate of diversity growth (diversity in one bin divided by the diversity in the previous bin) plotted against observed diversity, based on AICc-weight-averaged predicted values from models where K is controlled by each factor. General trends based on local regression fitting (LOESS) and their 95% prediction band are shown. Light grey represents the real trend (Fig. 1A). The second column shows the differences between AICc-weight-averaged predicted diversity from pure DD models and models where each factor regulates K, plotted against each factor. Points are colored according to the predicted diversity under each factor (darker blue is higher diversity). Dark lines represent linear or polynomial regressions that are significant ($P < 0.05$). Third and fourth columns are the same but here we average model predictions based on the influence of each factor on r_0.

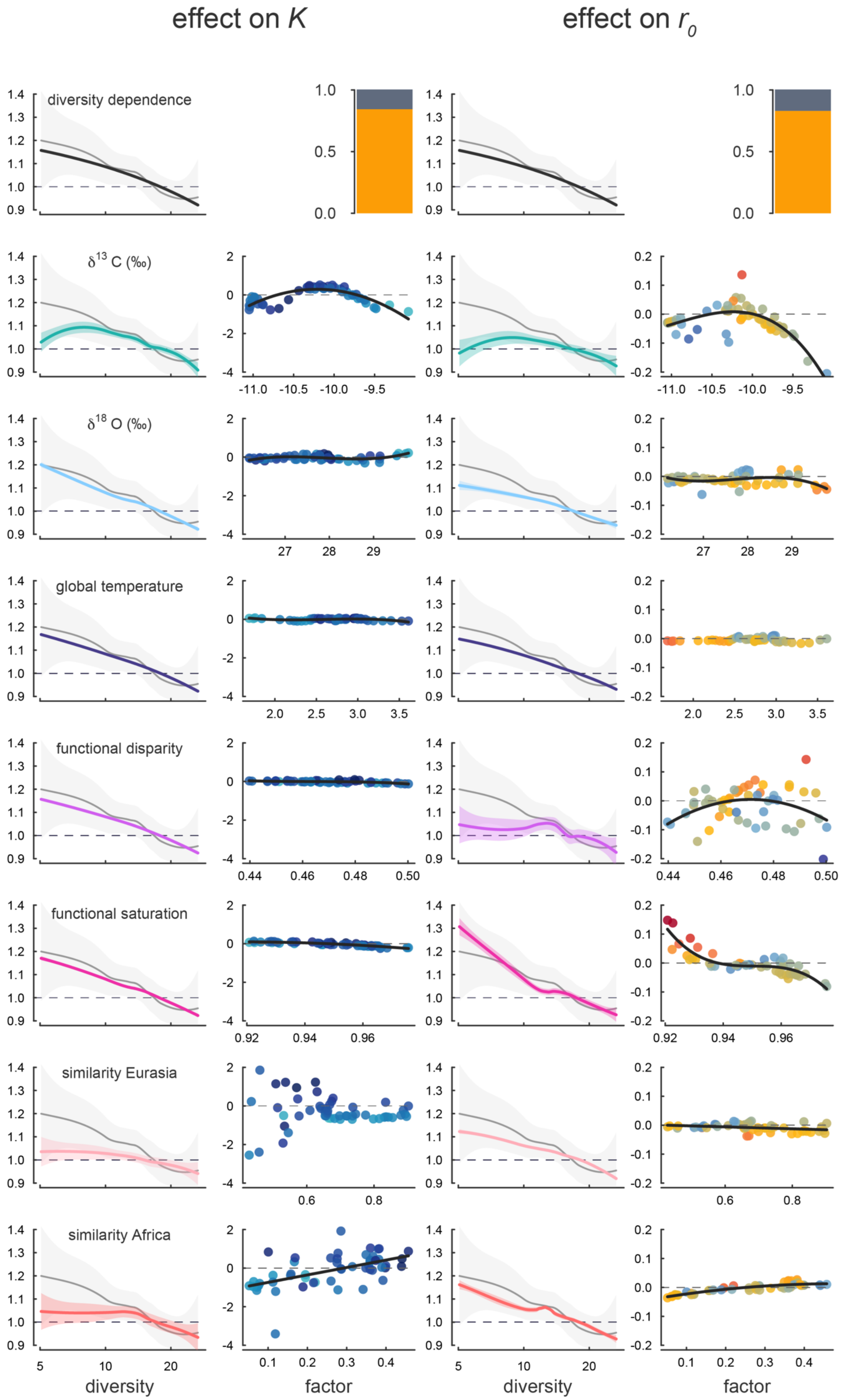

effect on K
effect on r_0
diversity dependence
δ^{13}C (‰)
δ^{18}O (‰)
global temperature
functional disparity
functional saturation
similarity Eurasia
similarity Africa
diversity
factor
diversity
factor

Figure S8. Results of model- averaging parameters from models based on TRiPS-estimated diversity. Bar-plots show the support for pure DD models in grey (fixed *K* and r_0, respectively). Effect of different factors on the strength of diversity-dependence. The first column shows the rate of diversity growth (diversity in one bin divided by the diversity in the previous bin) plotted against observed diversity, based on AICc-weight-averaged predicted values from models where *K* is controlled by each factor. General trends based on local regression fitting (LOESS) and their 95% prediction band are shown. Light grey represents the real trend (Fig. 1A). The second column shows the differences between AICc-weight-averaged predicted diversity from pure DD models and models where each factor regulates *K*, plotted against each factor. Points are colored according to the predicted diversity under each factor (darker blue is higher diversity). Dark lines represent linear or polynomial regressions that are significant ($P < 0.05$). Third and fourth columns are the same but here we average model predictions based on the influence of each factor on r_0.

Dataset S1. Information on the age of the fossil sites, and functional traits for the 209 mammalian species analyzed. Description of the 206 models run based on diversity curves estimated using TRiPS and SQS, showing classifications based on different criteria, AICc scores and AICc weights. Aggregated AICc weights for all the models are shown for each classification. Aggregated AICc weights based on AICc scores of the best model for each category in each classification.
